# Cellular Response upon Stress: p57 Contribution to the Final Outcome

**DOI:** 10.1155/2015/259325

**Published:** 2015-09-27

**Authors:** Marianna Nicoletta Rossi, Fabrizio Antonangeli

**Affiliations:** ^1^Department of Cellular Biotechnologies and Hematology, Pasteur Institute-Fondazione Cenci Bolognetti, Sapienza University of Rome, Viale Regina Elena 324, 00161 Rome, Italy; ^2^Department of Molecular Medicine, Pasteur Institute-Fondazione Cenci Bolognetti, Sapienza University of Rome, Viale Regina Elena 291, 00161 Rome, Italy

## Abstract

Progression through the cell cycle is one of the most important decisions during the life of a cell and several kinds of stress are able to influence this choice. p57 is a cyclin-dependent kinase inhibitor belonging to the CIP/KIP family and is a well-known regulator of the cell cycle during embryogenesis and tissue differentiation. p57 loss has been reported in a variety of cancers and great effort has been spent during the past years studying the mechanisms of p57 regulation and the effects of p57 reexpression on tumor growth. Recently, growing amount of evidence points out that p57 has a specific function in cell cycle regulation upon cellular stress that is only partially shared by the other CIP/KIP inhibitors p21 and p27. Furthermore, it is nowadays emerging that p57 plays a role in the induction of apoptosis and senescence after cellular stress independently of its cell cycle related functions. This review focuses on the contribution that p57 holds in regulating cell cycle arrest, apoptosis, and senescence after cellular stress with particular attention to the response of cancer cells.

## 1. Introduction

Cells can encounter different kinds of stress during their life and in turn have evolved a wide range of responses. Stress-activated signalling pathways such as ATM/ATR, JNK/SAPK, and p38 pathways are activated in mammalian cells by DNA damage, starvation, heat and osmotic shock, and oxidative stress. Depending on the kind, severity and duration of insult, and on the cell type, these responses can lead to different final outcomes, spanning from cell survival to cell death. Cell cycle delay or arrest is often the first safety step triggered in a stressed cell, followed by injury repair and thus restoration of cellular proliferation, or by the induction of cellular senescence or cell death.

Cellular senescence is defined as the irreversible exit from the cell cycle. In multicellular organisms there are three conditions in which cells stop dividing: quiescence, terminal differentiation, and senescence. Quiescence is reversible and it is usually induced by growth factor's withdrawal or contact inhibition, while in terminal differentiation and cellular senescence cell cycle arrest is permanent. During terminal differentiation cells acquire a distinctive phenotype and specialized functions in response to physiological stimuli. On the other hand, cells become senescent after exposure to peculiar types of stress [1]. Shortening of telomeres has been identified as the main stress inducing senescence in cultured cells* in vitro*, called for this reason replicative senescence. Genotoxic stress and more generally prolonged activation of the DNA damage response pathways results in the so-called premature senescence. Interestingly, cells usually arrest cell cycle in G1 phase during replicative senescence and in G2 phase during premature senescence. Senescent cells often display a flat, enlarged morphology and exhibit an increase in the lysosomal *β*-galactosidase activity that can be used as senescence biomarker (senescence-associated *β*-galactosidase activity or SA-*β*-gal activity). Many senescent cells also display a characteristic senescence-associated secretory phenotype (SASP) (for a review on cellular senescence see [2]). Senescence is thought to be a major barrier to tumor formation, as it limits the replicative potential of cells and seems to activate the immune system. Indeed, it has been reported that senescence limits the growth of many tumors including epithelial tumors of the colon, head and neck, and thyroid [3–5]. On the other hand, recent studies show that senescence is involved in tumor regrowth and disease recurrence, as senescent tumor cells can serve as a reservoir of secreted factors with mitogenic, antiapoptotic, and angiogenic activities [6].

Regarding cell death, different types of programmed cell death, including autophagy, apoptosis, and necroptosis have been described so far. Starvation is a canonical cellular condition that starts autophagy, but also damaged organelles are recycled by autophagy [7]. DNA damage, instead, represents a common type of cellular stress inducing apoptosis [8]. On the other hand, cells can undergo necroptosis, or necrosis-like caspase-independent programmed cell death, in presence of cellular inhibitor of apoptosis proteins (cIAPs) and caspase inhibitors [9].

Apoptosis is the most common type of programmed cell death by which the body eliminates damaged or exceeding cells without local inflammation. Accordingly, apoptosis plays several physiological and pathological roles, spanning from tissue remodelling during embryogenesis to cancer progression. Two main molecular pathways have been described so far, the so-called extrinsic and intrinsic pathways. The extrinsic pathway is triggered by the activation of death receptors located on the cellular membrane and is usually involved in processes of tissue homeostasis such as the elimination of autoreactive lymphocytes, while the intrinsic pathway is mainly mediated by the release of cytochrome *c* from mitochondria, a well-known cellular response to stress [10]. Both pathways lead to the activation of caspases, aspartate-specific cysteine proteinases, which mediate the apoptotic effects among which the cleavage of proteins responsible for DNA repair and cell shrinkage. Notably, many chemotherapeutic drugs kill cancer cells inducing apoptosis upon DNA damage or sensitize cancer cells to apoptosis to overcome drug resistance. To this regard, much effort has been spent to study and possibly control apoptosis in malignancies and so it is of fundamental importance to understand the molecular pathways and cellular conditions that regulate and trigger apoptosis. It is now clear, indeed, that drug/stress-induced damage can initiate different postdamage responses, including apoptosis and cellular senescence, depending on the balance of pro- and antiapoptotic factors and on the levels of regulators of the cell cycle [11, 12].

p57 (cyclin-dependent kinase inhibitor 1C or KIP2) is considered a master regulator of the cell cycle during embryogenesis and tissue differentiation [13, 14], but recently a broad spectrum of evidence indicates that p57 plays a role, sometimes distinct from cell cycle control, also in the cellular response to different stresses, regulating the induction of apoptosis and senescence. This review summarizes those findings with particular attention to the role that p57 plays in the cellular response to stress of cancer cells.

## 2. p57 Functions and Regulation

p57 belongs to the CIP/KIP family of cyclin-dependent kinase (CDK) inhibitors (CKIs) along with p21 and p27. The CIP/KIP family counteracts cell cycle progression inhibiting all the cyclin CDK complexes throughout the cell cycle (for a review on CKIs see [15]). In particular, p57 inhibits the complexes formed with CDK2, CDK3, and CDK4 and to a lesser extent CDK1 and CDK6 [16–18]. Induction of p57 causes cell cycle arrest mostly in G1 phase [16], even if cell cycle arrest in G2 phase has also been reported after p57 reinduction in cancer cells [19]. In addition to an N-terminal CDK inhibitory domain, homologs to the ones of p21 and p27, and a C-terminal QT-box, significantly homologous with that of p27, human p57 has a central domain rich in proline-alanine repeats responsible for additional p57 interactions, suggesting that p57 can exert different and/or more complex functions than its siblings [13, 14]. Indeed, the p57 internal domain has been reported to interact with the N-terminal of LIM domain kinase 1 (LIMK-1), a kinase involved in the control of actin dynamics, supporting the idea that the p57 internal domain may be responsible for p57 functions other than CDK inhibition [20, 21]. All the three members of the CIP/KIP family are evolutionary conserved among vertebrates, with p57 more closely related to p27 than p21, as indicated by the structural homology and phylogenetic relationship [22, 23].

Due to its role in cell cycle control, p57 is involved in the regulation of many cellular processes such as embryogenesis and tissue differentiation. In muscle differentiation p57 participates in the regulation of cell cycle exit of differentiating myoblasts [24, 25]. During hematopoiesis decreased levels of p57 are essential for the exit from quiescence and reentry into the cell cycle of hematopoietic stem cells [26–28]. p57 is also involved in the differentiation of several other cell phenotypes, including podocytes [29], placental cells [30], keratinocytes [31], pancreatic cells [32], hepatocytes [33], T-lymphocytes [34], and spermatozoa [35]. Other than differentiation, p57 seems to be involved also in controlling tissue aging. Indeed, Park and Chung analyzed the levels of p57 in muscle and lung during mouse aging showing that these levels go toward a clear decrease [36]. The mechanism of such a decrease was not investigated, but the authors suggested that p57 decrease could be associated to tissue aging as p57 can contribute to muscle differentiation and regeneration.

p57 is considered a tumor suppressor gene due to its ability to inhibit proliferation and the importance of p57 in the suppression of cancer is highlighted by its mutation/inactivation in the Beckwith-Wiedemann Syndrome, a cancer predisposing syndrome [37]. Remarkably, p57 expression is reduced in some human malignancies including lung cancer, hepatocellular carcinoma, and bladder cancer, confirming its involvement during tumorigenesis [38–40].

A cytoplasmic localization for p57 has been described in non-small-cell lung carcinoma and esophageal squamous cell carcinoma [41, 42], suggesting that p57 can exert different functions in different cellular compartments. Indeed, it seems that p57 is important for cytoskeletal dynamics and cell motility. As previously cited, it has been reported that p57 is able to bind LIMK-1, an enzyme that promotes actin filaments formation, and to sequester it into the nucleus [21]. Accordingly, the absence of p57 causes a delayed migration of neurons in the cortical plate during mouse development [43]. In contrast to these findings, Vlachos and Joseph confirmed in a human cervical adenocarcinoma (HeLa) cell line the interaction between p57 and LIMK-1, but they showed that this interaction does not result in the translocation of the kinase into the nucleus, but instead augments LIMK-1 activity, hence increasing actin-fiber formation [20]. Interestingly, also p21 seems to play different roles depending on cellular localization, as it can localize to the nucleus, where it regulates cell proliferation and differentiation, or in the cytoplasm, where it inhibits apoptosis [22, 44].

**Figure 1:**
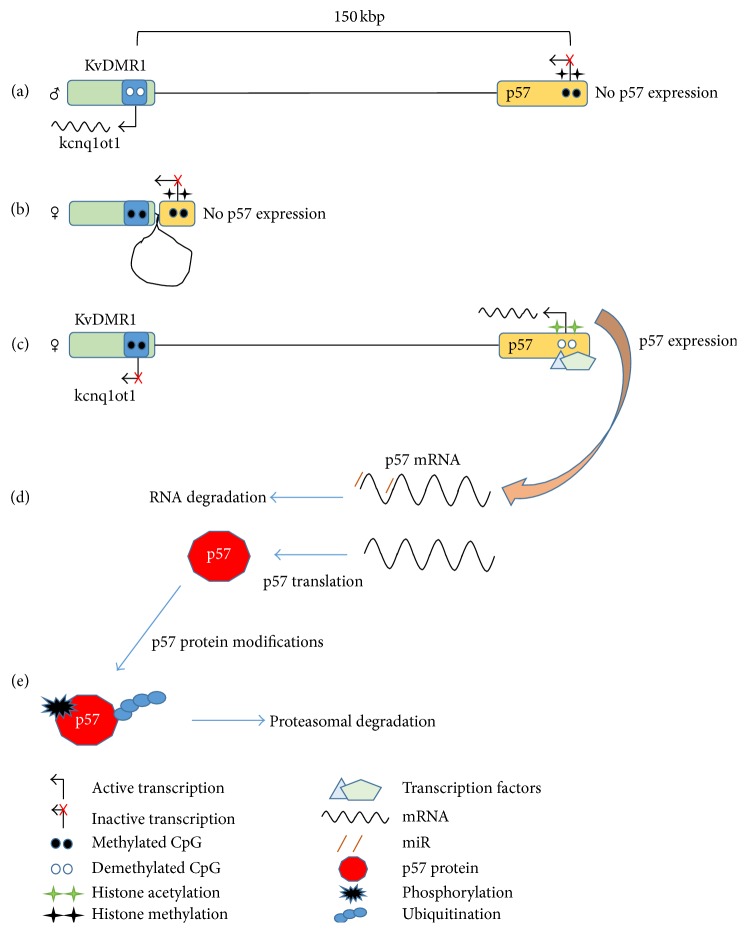
Different mechanisms of transcriptional and posttranscriptional p57 regulation. (a) Imprinting control on the paternal allele; (b) long-range intrachromosomal interactions between p57 promoter and KvDMR1 can repress the transcriptional expression; (c) promoter demethylation, active histone marks, and transcriptional factors binding regulate p57 expression; (d) p57 mRNA stability is regulated by miR; (e) p57 protein stability is regulated by phosphorylation and ubiquitination.

Considering that p57 is involved in many cellular processes, it is not surprising that it shows a complex regulation. The expression pattern of p57 has a highly specific spatial and temporal profile, reaching its peak and widespread distribution during embryogenesis and development, while in adults it is restricted to few tissues such as testis and muscle. The importance of p57 during embryogenesis emerges also from the analysis of the knockout mouse phenotype, as p57 null embryos present hyperplasia in several organs and cannot survive [14, 17]. The precise expression pattern of p57 is achieved by complex and multiple levels of transcriptional and posttranscriptional regulation (Figure 1). First of all, in both mouse and human, the p57 gene,* cdkn1c*, is located in the imprinted domain kcnq1/kcnq1ot1. Maternal and paternal allele of an imprinted gene display, despite the fact that they have identical sequences, different epigenetic modifications, such as DNA methylation within CpG islands, histones acetylation, and methylation. The imprinting of a cluster of genes is regulated by specific sequences acting in* cis*, known as imprinted control regions (ICR). In particular,* cdkn1c* is expressed only by the maternal allele, while the paternal allele is silent (Figures 1(a) and 1(c)) [14]. The imprinting regulation is achieved through the ICR KvDMR1, located 150 kbp downstream the p57 promoter. The repressive epigenetic status on the paternal allele is regulated by the long noncoding RNA kcnq1ot1, which is expressed only in the paternal allele from the ICR KvDMR1. Kcnq1ot1 is able to recruit the DNA methyl-transferase 1 and the histone methyl-transferases EZH2 and G9a on the promoters of imprinted genes, leading to the silencing of the paternal allele [45].

In addition to imprinting control,* cdkn1c* promoter harbors the binding sites for many transcription factors that regulate its expression in a cell type dependent manner (Figure 1(c)). For example, Sp1 and p73*β* are able to bind and induce* cdkn1c* promoter [46, 47], while Hes1, a Notch effector, suppresses the expression of p57 [48].

During muscle differentiation, another mechanism of transcriptional regulation has been described that involves p57 promoter long-range direct and functional association with the ICR KvDMR1 (Figure 1(b)). This association leads to the formation of a repressive intrachromosomal loop mediated by the insulator factor CTCF; this loop is destroyed during muscle differentiation by the binding of MyoD to the ICR KvDMR1 [49, 50].

Moreover, a series of microRNA (miR) have been reported to downregulate the expression of* cdkn1c* (Figure 1(d)). For example, miR-221 and miR-222 have been found overexpressed in many cancer types where they lead to p57 downregulation [51, 52].

Finally, p57 protein stability is regulated by both phosphorylation and ubiquitination (Figure 1(e)). In particular, p57 phosphorylation by different kinases leads to its ubiquitination and 26S proteasome-mediated degradation. CDK2-cyclin E complex phosphorylates p57 at Thr310 of the QT-box domain [53]; Akt, a kinase often deregulated in cancer, phosphorylates p57 at Thr310 or Ser282 [54]; while CHK1 (checkpoint kinase-1) phosphorylates p57 at Ser19 [55].

## 3. p57 at the Crossroad between Cell Cycle Arrest, Apoptosis, and Cellular Senescence

### 3.1. p57 Contribution to Cell Cycle Control upon Cellular Stress

**Figure 2:**
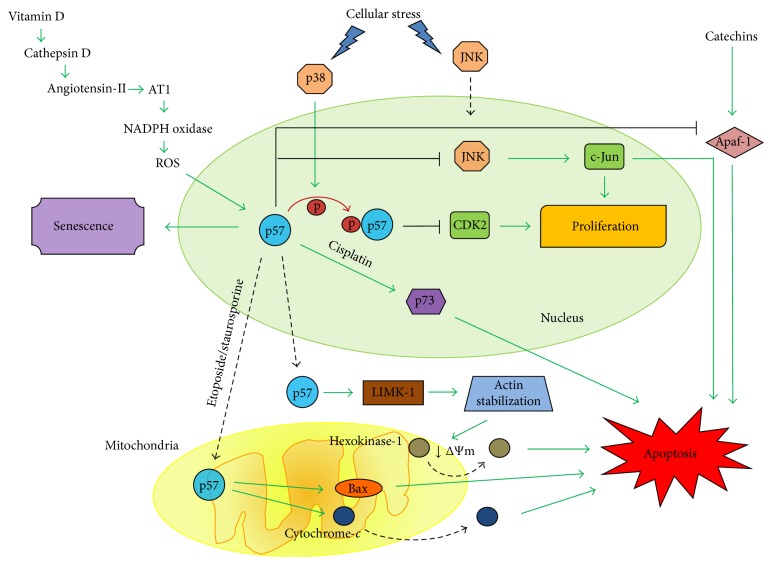
Network of p57 pathways involved in the cellular response to stress. Schematic view of the different mechanisms by which p57 can modulate proliferation, apoptosis, and senescence. Green arrows indicate positive regulation; black T-bar arrows indicate negative regulation; black dotted arrows indicate translocation; red arrow indicates phosphorylation; black facing-down arrow indicates loss of mitochondrial transmembrane potential (ΔΨm).

All the three members of the CIP/KIP family play an important role in controlling cell cycle exit. In particular, p57 is a master regulator of the cell cycle during embryogenesis and tissue differentiation [13, 14], but nowadays it is emerging that p57 has a specific function in cell cycle regulation upon cellular stress that is only partially shared by the other CIP/KIP inhibitors. Interestingly, this new role of p57 in controlling the cell cycle upon cellular stress has been reported to be both CDK inhibition-dependent and CDK inhibition-independent (Figure 2).

A CDK inhibition-dependent mechanism has been described for the stress-activated protein kinase (SAPK) p38 signalling, which is activated in mammalian cells by several insults, such as osmostress, oxidative stress, ionomycin, and UV [56–58]. It has been shown that SAPK p38 is able to phosphorylate p57 at Thr143 and this modification increases p57 affinity towards CDK2 resulting in cell cycle arrest at G1 in response to stress [59]. The increased activity of p57 is able to confer great resistance to different stimuli as cells lacking p38 or p57 display reduced viability to the previously cited stresses. Interestingly, phosphorylation of p57 by p38 neither affects its stability nor its localization, highlighting a novel mechanism of action of p57 after stress different from that observed during cellular differentiation that instead involves p57 induction/degradation. Notably, while hematopoietic stem cells that lack p57 present elevated levels of p27, suggesting that maintenance of cell quiescence is a common feature of p57 and p27, embryonic fibroblast knockout for p57 subject to stress are not able to increase p27, confirming that response to stress is a peculiar role of p57 [59].

On the other hand, p57 participates in the c-Jun NH_2_-terminal kinase/stress-activated protein kinase (JNK/SAPK) pathway with a CDK inhibition-independent mechanism. Indeed, p57 negatively regulates the JNK/SAPK signalling cascade through direct inhibition of JNK/SAPK, independently of its well-known inhibitory function on CDKs [60]. Deletion mutant experiments showed that p57 inhibits JNK and CDK2 by distinct mechanisms. In particular, p57 interacts with JNK1 through its QT-BOX domain and this interaction is able to preclude the interaction between JNK1 and c-Jun [60], while the inhibition of CDK2 is achieved through the CDK inhibitory domain. JNK/SAPK activation is implicated in the regulation of different cellular activities ranging from cell growth to cell death [61, 62]. Previous studies using p57 knockout mice reported an increase in apoptosis and altered differentiation during mouse development [63, 64]. It is plausible, therefore, to conclude that JNK/SAPK inhibition could be an important mechanism by which p57 exerts its functions not only on proliferation but also on cell death. Notably, this antiapoptotic role of p57 mediated by JNK/SAPK inhibition is in sharp contrast to the proapoptotic effects of p57 overexpression in cancer cells highlighted in the following section of this review.

### 3.2. p57 and Apoptosis

p21 and p27, the other members of the CIP/KIP family, have been reported to play a role in apoptosis [65, 66] and it is now emerging that p57 is implicated too (Figure 2). Samuelsson and colleagues reported that p57 expression enhances apoptosis in HeLa cells treated with staurosporine, a protein kinase C inhibitor, and showed that p57 itself is a target of caspase activity [67]. In contrast to p27, which has a proapoptotic activity by itself [68], p57 was found to have only a minor proapoptotic effect on its own, but rather to sensitize cells to apoptosis. In agreement, a concomitant expression of the antiapoptotic factors Bcl-x_L_ or Bcl-2 was found to counteract p57 proapoptotic effect. The molecular mechanism by which p57 promotes cell death was investigated by Vlachos and colleagues [69]. p57 overexpression is able to sensitize cancer cells to apoptotic agents such as cisplatin, etoposide, and staurosporine via a mechanism that is independent of p57 ability to inhibit CDK activity in the nucleus. In particular, p57, within minutes from drug treatment, translocates into mitochondria promoting Bax activation and loss of mitochondrial transmembrane potential, thus triggering the intrinsic apoptotic pathway through the cytochrome *c* release into cytosol and consequent caspase-9 and caspase-3 activation. Mitochondrial pathway specificity was confirmed, as p57 expression was ineffective in promoting death receptor-mediated apoptosis stimulated with agonistic anti-FAS antibodies. The mechanism by which p57 triggers the mitochondrial intrinsic pathway has been linked to the ability of p57 to stabilize the actin cytoskeleton by Kavanagh and colleagues [70]. They demonstrated that p57 directly interacts with LIM domain kinase-1 (LIMK-1) resulting in an increase in LIMK-1 kinase activity, which is required for both p57-mediated actin cytoskeleton stabilization and p57 death promoting effect. Indeed, LIMK-1 is able to inactivate the cytoskeleton remodelling factor cofilin that is involved in the disassembling of actin filaments and it has already been supposed that stabilization of the actin cytoskeleton can promote apoptotic cell death [71]. Furthermore, p57-mediated stabilization of actin leads to the displacement of hexokinase-1, an inhibitor of the mitochondrial voltage-dependent anion channel, from mitochondria, providing a possible mechanism for mitochondrial depolarization and therefore for the promotion of the mitochondrial apoptotic cell death pathway.

p57 proapoptotic effects were observed in different cell lines, including HeLa cervical cancer cells, SH-SY5Y neuroblastoma cells, SKOV3 ovarian carcinoma cells, and mouse embryonic fibroblast cells [69]. In H1299 lung cancer cells and HCT116 colorectal carcinoma cells, silencing of p57 was shown to suppress p73*β*-mediated apoptosis induced by cisplatin treatment [72]. However, the death promoting effect of p57 was not seen in HEK-293 human embryonic kidney cells, suggesting some degree of cell type specificity, likely due to the balance between survival and death pathways that are active in a particular cell type.

Kuang and colleagues performed an interesting study on different leukemia cell lines that differ for p57 promoter methylation status [19]. They analyzed the expression of p57 after different stimuli such as transforming growth factor-*β*, lipopolysaccharide, tumor necrosis factor-*α*, insulin-like growth factor 1, and different forms of cellular stress such as high-density culture or serum withdrawal. They found p57 reactivation only in cell lines with unmethylated promoter but not in methylated cells, leading to different outcome ranging from no effect on cell growth to G2 arrest and apoptosis, depending on cell type and type of insult. Remarkably, exogenous overexpression of p57 in p57 promoter-methylated leukemic cell lines resulted in marked cell growth arrest and induction of apoptosis, while the overexpression of p57 in partially methylated cells only resulted in a moderate inhibition of cell growth and had no impact on apoptosis, suggesting that the epigenetic status of p57 promoter can influence the cell response to stress stimuli.

It is worthy of notice that the proapoptotic effects of p57 described so far have been mainly reported in cancer cells after p57 overexpression or reinduction, while antiapoptotic activity of p57 has been observed in its physiological regulation of JNK pathway (see previous section) and during embryogenesis, suggesting that cellular context drives a major contribution to the final outcome. For example, antiapoptotic activity of p57 was reported in response to green tea polyphenols administration. Catechins are known to induce cell death in many types of tumor cells, but normal human epithelial cells were found to survive in the presence of polyphenols because of their ability to induce p57 expression. p57 was able to prevent green tea polyphenols-induced Apaf-1 expression thus avoiding apoptosis [73]. Finally, p57 has been suggested to have an antiapoptotic role in the gastrointestinal tract and the lens of the eye during embryonic development [63, 74].

### 3.3. p57 and Cellular Senescence

In two human hepatocarcinoma cell lines (HepG2 and SNU398) p57 overexpression has been found to affect proliferation and morphology without affecting the apoptotic machinery. In these cells, p57 expression is regulated by neither miR-221 nor the methylation status of the promoter but instead by the Notch target gene* Hes1*. p57 infected cells or Notch1- and Notch3-silenced cells, which upregulates p57, arrest growth with a senescent morphology, SA-*β*-gal staining, and p16 expression [75].

In a similar manner, Tsugu and colleagues have shown that p57 induction in p57-negative human astrocytoma cell lines (U343, U87, and U373) can block the proliferation and alter the morphology, with cells becoming large and flat with an expanded cytoplasm [76]. These flat cells resemble the senescent phenotype even if the SA-*β*-gal activity was reported to be partially reversible withdrawing p57 forced expression. Although senescent cells are thought to be resistant to apoptotic cell death, in one of the astrocytoma cell lines induced to express p57 (U373), a small subset of cells (15% of the population) was described to undergo apoptosis. Notably, Bax levels were unchanged. Why this occurs and why this particular cell line responds in a different manner to p57 induction is an interesting yet unanswered question.

A pivotal role of p57 in the premature senescence of vascular smooth muscle cells has been shown by Valcheva and colleagues in vitamin D receptor knockout mice [77]. Interestingly, they show a direct link between oxidative stress, p57 induction, and the onset of the senescent phenotype (Figure 2). Indeed, lack of vitamin D signalling results in increased cathepsin D enzymatic activity, which in turn augments angiotensin-II production. The binding of angiotensin-II to its receptor AT1 increases NADPH oxidase activity and free radical production. The latter induces high levels of p57 that trigger the premature senescence of vascular smooth muscle cells. Senescent vascular smooth muscle cells have been found in atherosclerotic plaques [78] and recent results suggest that vascular smooth muscle cell senescence could even promote atherosclerosis [79], tempting to speculate that p57 could become a therapeutic target. More studies are needed to deepen the interesting correlation between ROS production and p57 increase.

## 4. Concluding Remarks

Initially identified as a cyclin-dependent kinase inhibitor, p57 has since been shown to have different cellular roles aside from cell cycle inhibition, such as cell migration and regulation of cell differentiation. Nowadays it is emerging that p57 plays a key role also in coordinating the cellular response to stress, being able to drive to both apoptosis and cellular senescence. Different mechanisms of action, both CDK inhibition-dependent and CDK inhibition-independent, have been disclosed and, as highlighted in this review, p57 is now implicated in the crosstalk between several different pathways, among which MAPK signalling, DNA damage response, mitochondrial apoptotic pathway, and cytoskeleton organization. The findings that p57 can induce cell cycle arrest, apoptosis, or cellular senescence depending on cell types and cellular context arise several questions:

- Is the final outcome dependent on p57 levels?
- As many data come from* in vitro* studies and overexpression of any gene can lead to experimental artefacts, which is the physiological relevance of p57 induction* in vivo*?
- Which is the grade of overlapping between the three members of the CIP/KIP family?
- Bearing in mind that stopping abnormal proliferation is a key goal of our scientific community, is the reinduction of p57 a promising approach for cancer therapy?
- Do cancer cells respond in a different way from normal cells to p57 overexpression?

p57 is now emerging as a new master regulator of cell fate and the mechanisms through which p57 participates in the cellular response to stress have been just started to be dissected.
